# Rhizosphere Microbiome Engineering for Climate-Smart Agriculture: From Synthetic Consortia to Precision Decision Support

**DOI:** 10.3390/microorganisms14051138

**Published:** 2026-05-17

**Authors:** Nourhan Fouad, Emad M. Elzayat, Dina Amr, Dina A. El-Khishin, Khaled H. Radwan, Alaa Youssef, Abeer A. Khalaf, Hoda A. Ahmed, Eman H. Radwan, Sawsan Tawkaz, Michael Baum

**Affiliations:** 1 International Center of Agricultural Research in Dry Areas (ICARDA), Giza 11742, Egypt; n.mahmoud@cgiar.org (N.F.); alaayosuf1@gmail.com (A.Y.); s.tawkaz@cgiar.org (S.T.); m.baum@cgiar.org (M.B.); 2 Department of Biotechnology, Faculty of Science, Cairo University, Giza 12613, Egypt; elzayatem@sci.cu.edu.eg (E.M.E.); dina@sci.cu.edu.eg (D.A.); 3 Agricultural Genetic Engineering Research Institute (AGERI), Agricultural Research Center (ARC), Giza 12619, Egypt; dina_elkhishin@yahoo.com (D.A.E.-K.); abeerahmedik@gmail.com (A.A.K.); 4 Department of Biological Sciences, College of Science, King Faisal University, Al Ahsa 31982, Saudi Arabia; haahmed@kfu.edu.sa; 5 Zoology Department, Faculty of Science, Damanhour University, Damanhour 22511, Egypt; eman.radwan@sci.dmu.edu.eg

**Keywords:** biocontrol, nutrient use efficiency, microbiome formulation, strain tracking, multi-omics, precision agriculture

## Abstract

Rhizosphere microbiome engineering is a promising approach that can enhance crop resilience and input use efficiency by redirecting plant–microbe–soil interactions toward predictable functions. Here, we review the mechanistic bases underlying rhizosphere assembly and stability, including root exudate-mediated selection, priority effects, keystone taxa, and metabolite-driven signaling, and connect these principles to proposed design rules for microbial inoculants. We present a generalizable Design–Build–Test–Learn (DBTL) framework for engineering synthetic microbial consortia, covering trait-to-module mapping (nutrient acquisition, phytohormone modulation, ACC deaminase activity, stress-protective metabolites, and biocontrol), compatibility screening, minimal yet robust community architectures, and iterative optimization driven by multi-omics and high-throughput phenotyping. Translation to field settings is framed as an engineering challenge defined by formulation and administration limitations, including carrier type, seed coating and encapsulation methods, shelf life, strain invasiveness, and permanence of colonization amid environmental diversity. We also summarize how integrative measurement pipelines (amplicon and shotgun sequencing, transcriptomics, metabolomics, and network or causal analyses) can advance microbiome studies from correlation to actionability. We describe how precision agriculture (sensors, remote sensing, and variable-rate inputs) and AI/ML (split-sample comparisons, transfer learning, and active learning) approaches can accelerate strain discovery, mixture optimization, and adaptive experimentation, driven by the need for stringent controls, metadata-rich reporting, and cross-site comparability. Use cases focus on stress conditions (drought, salinity, thermal extremes, and biotic stress) to demonstrate how microbial functions translate to agronomic outcomes and to highlight critical bottlenecks for reproducible, scalable microbiome products.

## 1. Introduction

Engineering the rhizosphere microbiome is a transformative approach in 21st century agriculture for addressing the complex challenges of sustainable global food production. The soil surrounding plant roots, known as the rhizosphere, is a promising microbiological frontier because it harbors unique biodiversity and serves as a dynamic interface where plants, soil, and microbes interact. This ecosystem is influenced by various biotic factors, such as plant species and microbial community composition, and abiotic factors, such as soil type and moisture, which together support plant health and productivity [1,2].

The conceptual framework for agricultural sustainability is based on the intersection of plant–soil–microbe relationships. Microbial communities in the rhizosphere can regulate the growth dynamics of plant-beneficial microbes involved in nutrient cycling, disease suppression, and increased plant resilience to stress [3,4,5]. Certain microbes, including plant-growth-promoting rhizobacteria (PGPR) and mycorrhizal fungi, promote plant health either directly (e.g., through nutrient acquisition) or indirectly (e.g., by improving soil structure) [6,7].

Historically, research on plant–microbial interactions was largely descriptive, and management of these interactions was mostly reactive rather than design-driven. More recently, however, the field has been moving in an increasingly accelerated, design-oriented direction. Plant performance, such as drought resistance and nutrient uptake, can be improved by tuning community structure with synthetic microbial consortia [8,9,10]. To support this transition, eco-evolutionary principles must be rigorously applied to provide a framework for creating engineered systems capable of recapitulating the functional landscape of native microbiomes [11,12].

Compared with earlier reviews that focus primarily on plant growth-promoting microbes, synthetic communities, or plant microbiome engineering in general, this review provides an integrated framework connecting rhizosphere ecology, consortium design, formulation science, multi-omics, AI-enabled optimization, and field validation within the context of climate-smart agriculture. Instead of treating these themes separately, we organize them as linked stages in a translational pipeline from ecological principles to deployment and decision support. We also place greater emphasis on constraints, reproducibility, field realism, and comparative evaluation of intervention strategies than is typical in broader descriptive reviews.

This review synthesizes the current state of rhizosphere microbiome engineering (RME) for researchers, agronomists, and decision-makers involved in sustainable crop production. It covers rhizosphere ecology, consortium design, formulation and delivery, multi-omics, AI-assisted optimization, and field translation in agriculture. Table 1 provides a summary of the technologies and strategies discussed.

Constructing microbial consortia systematically and methodically, along with developing a thorough understanding of ecological interactions in the rhizosphere, creates opportunities for advances in precision agriculture, improving crop production efficiency and addressing environmental challenges.

### Microbiome Engineering and Climate-Relevant Soil Functions

Beyond crop performance, rhizosphere microbiome engineering may influence climate-relevant soil functions. Improved nitrogen acquisition and better synchronization between plant demand and microbial nutrient turnover may reduce fertilizer losses and increase fertilization efficiency, particularly when microbial interventions enhance nutrient mobilization, rhizosphere activity, and plant nutrient uptake efficiency [4,10]. Microbiome-mediated changes in nitrification and denitrification could also affect N_2_O emissions, although the magnitude and direction of these effects remain highly context-dependent and require field verification under realistic agronomic conditions [4,5].

Carbon-related effects are also plausible: root-associated microbes can alter rhizodeposition, stimulate biofilm formation, improve aggregate stability, and reshape microbial carbon processing, thereby influencing pathways of soil carbon retention and stabilization. Rhizodeposition is increasingly recognized as a major contributor to the formation of stable soil organic matter fractions, particularly mineral-associated organic carbon, while microbial extracellular polymeric substances and biofilm-associated processes can improve aggregate formation and the physical protection of carbon in soil [13,14]. In stress-prone systems, these processes may be especially relevant because microbially mediated root inputs, aggregate stabilization, and carbon turnover can influence how carbon is retained under drought or saline conditions.

Consistent with this, Ref. [15] showed that amendment-driven shifts in bacterial community composition, soil aggregate stability, and soil organic carbon sequestration were closely linked in saline–alkali soils, supporting a mechanistic connection between microbial community change and carbon stabilization under conditions relevant to climate-smart agriculture. However, these mitigation-related claims should still be interpreted cautiously because direct long-term field evidence linking engineered rhizosphere consortia to durable reductions in N_2_O emissions or sustained soil carbon sequestration remains limited.

## 2. Rhizosphere Fundamentals

As an important ecological boundary, the rhizosphere serves as the central interface for interactions between plants and soil microorganisms. Understanding the fundamentals of this ecosystem is essential for developing strategies to design microbiomes that enhance agricultural productivity and sustainability.

**Figure 1:**
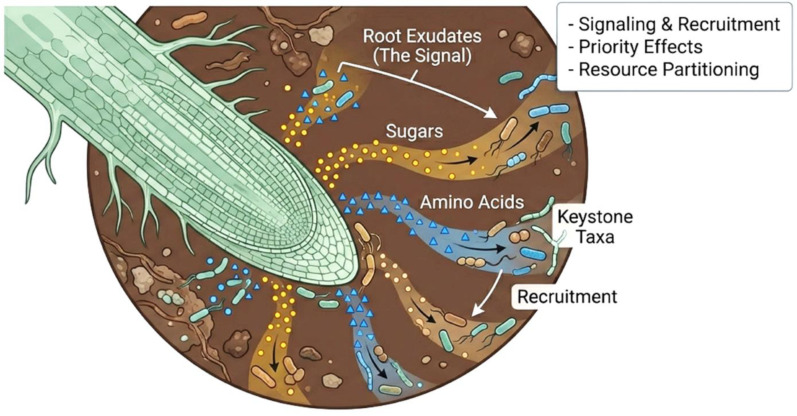
Root exudates as ecological currency: major exudate classes (sugars, amino acids, organic acids, and phenolics) and their roles in shaping rhizosphere community assembly, signaling, nutrient mobilization, and disease suppression.

As key mediators of rhizosphere interactions, root exudates can be considered ecological currency and are crucial for plant–microbe communication and interactions. These exudates include sugars, amino acids, and other organic compounds, such as organic acids and phenolics, which are released into soil environments [16,17]. The chemical nature of root exudates can influence microbial communities by preferentially selecting beneficial taxa that stimulate plant growth and health [18]. Differences in exudate profiles have been shown to favor certain microbial assemblages involved in disease suppression and nutrient provision [19,20]. Variation in root exudation among plant species may induce competition for microbial resources, affecting nutrient cycling and ecosystem processes [21]. Additionally, spatial and temporal changes in root exudate composition, directly influenced by environmental factors, can affect the structure of rhizosphere microbial communities [22,23] and, consequently, the maintenance of plant–microbe interactions. Key exudate classes and their downstream ecological effects are summarized in Figure 1.

Molecular mechanisms linking microbial community assembly in the rhizosphere to higher-order ecological interactions shaped by priority effects and keystone taxa govern community stability. Priority effects occur when the first microbes colonizing the rhizosphere determine the trajectories of later-colonizing microbial communities, which subsequently influence ecosystem functions [24]. Keystone taxa are species that have a disproportionate influence on community dynamics and stability [25]. For example, various bacteria and fungi can act as keystone species involved in nutrient cycling or disease resistance [26,27]. Previous studies have indicated that concentrating the ecological principles of stability and resilience in keystone species is a prerequisite for engineering microbial consortia [28,29].

This approach will aid in designing novel, effective microbial interventions that can modulate and tolerate various stressors in dynamic environments. Microbial interactions with plants occur mainly through signals and metabolites. Root exudates can recruit beneficial microbes, signal resource availability, and influence competitive interactions in the rhizosphere [30,31,32,33,34]. Understanding these signaling processes is important for rational microbiome manipulation and the development of interventions that improve crop resilience and yield [35,36].

A deeper understanding of the mechanisms of rhizosphere interactions, factors governing root exudation, community assembly, and signaling pathways or communicative processes will be essential for the rational design of intervention strategies to enhance plants and improve food productivity.

### Conceptual Limits and Context Dependency in Rhizosphere Microbiome Engineering

Although root exudate-mediated selection, priority effects, and keystone taxa are often presented as complementary mechanisms governing rhizosphere assembly, their relative importance is highly context-dependent. Root exudate chemistry can influence microbial substrate preference and succession in the rhizosphere, but exudate composition varies with plant genotype, developmental stage, nutrient status, and environmental conditions [16]. Similarly, priority effects can shape community assembly when early colonizers modify resource availability or niche conditions, but their persistence depends on dispersal, resident community structure, and environmental filtering [37,38].

A useful conceptual framework is to view rhizosphere assembly as a sequence of ecological filters. The first filter is the regional and soil microbial species pool. The second is the physicochemical environment, including soil texture, pH, organic matter, moisture, salinity, nutrient availability, and land-use history. The third is host-mediated selection through root architecture, exudation, and immune signaling. The fourth is microbial interaction, including competition, facilitation, antagonism, niche pre-emption, and priority effects. Evidence from plant microbiome studies indicates that selection, dispersal, drift, and diversification interact to structure plant-associated microbial communities rather than operating as a single deterministic process [37,39].

Thus, current theories are not necessarily irreconcilable but instead operate at different ecological scales. Root exudate-mediated selection explains host influence; priority effects explain colonization history; keystone taxa explain disproportionate functional influence; and resilience theory explains persistence after disturbance. The challenge for microbiome engineering is to identify when these mechanisms align and when they conflict. In practical terms, rhizosphere microbiome engineering should be framed less as deterministic control and more as probabilistic steering of microbial functions under defined crop–soil–climate constraints [10,40]. For example, host-driven recruitment may appear dominant in simplified or controlled systems, whereas in field soils, resident community composition and physicochemical filtering may override host effects, leading to different conclusions across studies.

In this review, keystone taxa are defined as taxa that have a disproportionate influence on community structure or function relative to their abundance. Stability refers to the maintenance of composition and/or function over time. Resilience refers to a community’s capacity to recover function after disturbance. Engineering is used here to mean intentional, evidence-based steering of microbiome structure or function under defined constraints, rather than deterministic control.

## 3. Consortium Design: From Wish List to Bill of Materials

**Figure 2:**
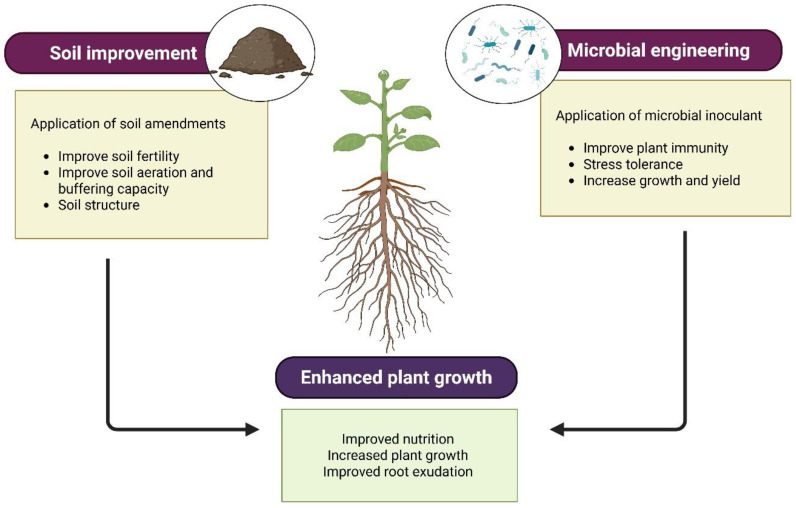
From ‘wish list’ to bill of materials: a workflow for rational consortium design linking target traits, functional modules, candidate taxa, compatibility screening, and iterative optimization.

Engineering the rhizosphere microbiome by designing specific microbial consortia is a key strategy for promoting plant health and sustainable agriculture. The combination of functional roles, interactions among constituent organisms, and a design based on ecological resilience and capacity are all important for an effective consortium. An end-to-end workflow for rational consortium design is outlined in Figure 2.

### 3.1. Functional Roles

To ensure successful plant development and abiotic stress tolerance, microbial consortia can be designed to meet specific functional needs. These consortia play essential roles in nitrogen fixation, phosphorus and potassium mobilization, phytohormone production, 1-aminocyclopropane-1-carboxylate (ACC) deaminase production, and pathogen biocontrol. Nitrogen-fixing bacteria, including those from the genera *Rhizobium* and *Azospirillum*, are important for increasing nitrogen bioavailability, while mycorrhizal fungi enhance phosphorus supply [41]. Additionally, microbial production of phytohormones such as auxins and gibberellins can promote plant growth and increase tolerance to biotic and abiotic stresses [42,43]. Key biocontrol agents further support plant health by suppressing pathogens. Isolating and assembling these functional groups into consortia tailored to specific crops can improve nutrient uptake, plant health, and yield. Table 2 summarizes the key functional modules, representative taxa of functional groups, and anticipated plant benefits.

Beyond nitrogen fixation, nutrient solubilization, phytohormone production, ACC deaminase activity, and biocontrol, several other functional traits should be considered during consortium design. Hydrolytic enzymes such as chitinases, cellulases, proteases, lipases, and β-1,3-glucanases contribute to pathogen suppression by degrading fungal cell wall components and interfering with pathogen establishment [44,45]. Exopolysaccharide-producing bacteria can enhance soil aggregation, rhizosphere hydration, biofilm formation, and tolerance to drought or salinity, making EPS production especially relevant for dryland and saline agriculture [44,46]. Some rhizosphere bacteria may also produce or modulate abscisic acid-related signaling, influencing stomatal regulation, stress-responsive gene expression, drought adaptation, and responses to water excess or flooding [40,46]. For field deployment, candidate strains should also be screened for tolerance to commonly used pesticides and agrochemicals, as chemical incompatibility may reduce survival and efficacy after application.

**Table 2:** Functional roles of key microbial groups in rhizosphere engineering.

Functional Role	Representative Microbial Taxa (Examples)	Mechanism in the Rhizosphere	Expected Plant Benefit	Relevant Environmental Condition(s)	Scope of Activity/Deployment Context	Key References
**Symbiotic and non-symbiotic nitrogen fixation**	*Rhizobium*, *Bradyrhizobium*, *Azospirillum*, *Azotobacter*, *Paenibacillus*	Biological conversion of N_2_ to NH_4_^+^ via nitrogenase	Improved N nutrition, biomass accumulation, and yield	Low-N soils; low-input systems; drought-prone systems where N uptake is constrained	Nutrient support; partial reduction in fertilizer dependence; best suited to systems where N limitation is a major constraint	[47,48,49,50,51]
**Phosphate solubilization**	*Pseudomonas*, *Bacillus*, *Enterobacter*, *Burkholderia*	Secretion of organic acids, phosphatases, and phosphonate-transforming enzymes	Enhanced P availability, root growth, and early vigor	P-deficient soils; calcareous or P-fixing soils	Nutrient mobilization; can complement fertilization programs rather than fully replace them	[52,53,54]
**Potassium mobilization**	*Bacillus mucilaginosus*, *Frateuria aurantia*, *Paenibacillus* spp.	Mineral weathering and release of K through acidification and chelation	Improved nutrient balance, osmotic adjustment, and stress tolerance	K-deficient soils; salinity- or drought-affected systems	Nutrient support in marginal soils; often used as part of multi-trait inoculants	[55,56,57]
**Phytohormone production**	*Azospirillum*, *Serratia*, *Burkholderia*, *Bacillus* spp.	Production of IAA, gibberellins, cytokinins, and related signaling compounds	Enhanced root architecture, shoot growth, and stress responsiveness	Early growth stages; stress-prone environments; transplant establishment	Growth promotion and developmental modulation; strongest effects are often observed when coupled with nutrient or stress mitigation traits	[58,59,60]
**ACC deaminase activity**	*Pseudomonas*, *Variovorax*, *Enterobacter* spp.	Degradation of ACC, lowering stress ethylene levels	Improved tolerance to drought, salinity, flooding, and transplant shock	Drought, salinity, waterlogging, and combined abiotic stress	Stress mitigation; particularly relevant in climate-variable systems	[61,62,63]
**Siderophore production**	*Pseudomonas fluorescens*, *Bacillus subtilis*, *Streptomyces* spp.	Chelation of Fe^3+^, improved Fe acquisition, and competition with pathogens	Better iron nutrition and indirect disease suppression	Fe-limited soils; saline soils; pathogen-prone rhizospheres	Nutrient acquisition plus indirect biocontrol; useful in multifunctional consortia	[64,65,66,67]
**Biocontrol/ISR induction**	*Trichoderma*, *Bacillus*, *Pseudomonas*, *Streptomyces*	Antibiotics, lipopeptides, volatile compounds, competition, ISR induction	Reduced soil-borne diseases and improved plant health	Disease-prone soils; high pathogen pressure; stress-weakened crops	Biotic stress management; often crop- and pathosystem-specific	[51,67,68]
**Hydrolytic enzyme production**	*Bacillus*, *Pseudomonas*, *Streptomyces*, *Trichoderma*	Production of chitinases, glucanases, cellulases, proteases, and lipases that degrade pathogen cell walls	Pathogen suppression and improved rhizosphere competitiveness	Biotic stress; fungal disease pressure; residue-rich soils	Biocontrol and pathogen suppression; useful where direct antagonism is required	[44,45]
**Exopolysaccharide (EPS) production/biofilm formation**	*Azotobacter*, *Bacillus*, *Pseudomonas*, *Rhizobium*	EPS-mediated biofilm formation, aggregation of soil particles, improved water retention, and root adhesion	Improved drought/salinity tolerance, rhizosphere stability, and persistence	Drought, salinity, coarse-textured soils, fluctuating moisture	Stress buffering and colonization support; valuable for field persistence and dryland systems	[44,45,46]
**ABA modulation/production**	*Azospirillum*, *Bacillus*, *Pseudomonas* spp.	Modulation of ABA-related signaling and stress-responsive gene expression	Improved stomatal regulation and adaptation to drought, excess moisture, or fluctuating water status	Drought, flooding transitions, unstable rainfall regimes	Stress-signaling support; still less standardized than ACC deaminase-based selection	[40,46]
**Pesticide/agrochemical tolerance**	Stress-adapted PGPR and fungi from managed agroecosystems	Survival and function under seed-treatment chemicals, pesticides, and mixed input regimes	Improved persistence and more reliable field performance	Conventional farming systems with seed treatments or pesticide use	Deployment compatibility; critical for commercial translation and realistic field adoption	[40,69]

### 3.2. Rules of Engagement

The interactions between the constituent species must be understood to effectively engineer a microbial consortium. Even so, compatibility matrices can help identify positive synergies with fewer negative interactions [70,71]. Certain bacteria, for example, secrete allelochemicals that suppress the growth of pathogenic or competing microbial species, thereby promoting beneficial species. Conversely, if species compete for substrates or produce toxic metabolites, the consortium’s activity decreases. By employing a systematic screening method to assess interactions and cohabitation in microbial networks, the compositional compatibility and ecological stability of synthetic communities can be ensured, thereby promoting performance [72].

### 3.3. Minimal Yet Resilient Consortia

While interest in the functional composition of consortia has suggested that small but diverse microbial communities tend to deliver the best function and stability [73,74], recent studies have revealed a more complex picture. The benefits of small consortia are compelling, but there are diminishing returns from larger consortia; as complexity and broader interactions increase, the returns from alliances can become negative. Identifying this “sweet spot” can lead to niche partitioning, allowing microbes to utilize resources more efficiently and increasing community stability [75,76]. The underlying science is based on biodiversity concepts, which advocate selecting only as many species as the available niches can sustain—that is, species that can play complementary roles within the same community.

### 3.4. Synthetic Ecology Playbook

DBTL provides an iterative framework for engineering microbial consortia. It is a continuous, iterative process in which hypotheses about microbial behavior, collective interactions, and functions are experimentally tested. Consortia are generated, evaluated, and refined based on these findings, allowing for ongoing improvement [77,78]. This iterative engineering approach enables rapid adaptation and innovation for synthetic ecologists. Integrating omics technologies within the DBTL framework allows for detailed characterization of microbial behavior, interaction functions, and consortium dynamics, providing a foundation for microbiome engineering in crop management [42,43].

**Figure 3:**
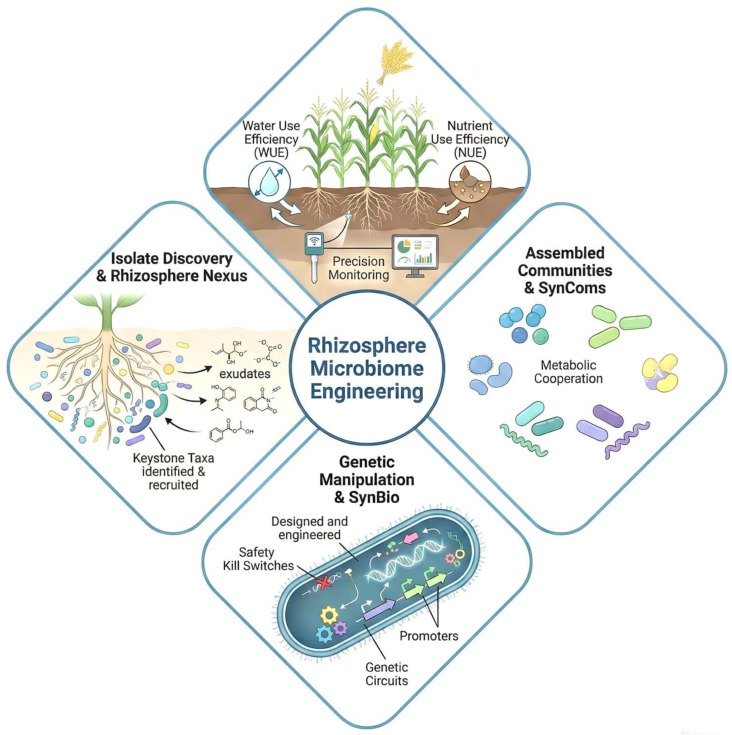
Design–Build–Test–Learn (DBTL) loop for synthetic rhizosphere consortia, integrating omics, phenotyping, and field feedback to refine strain selection, ratios, and delivery conditions.

New microbial consortia should be designed around functional roles, compatibility, and optimal composition to support effective and resilient deployment. Figure 3 illustrates the iterative DBTL cycle used to refine consortium composition and deployment.

### 3.5. Worked DBTL Example: Drought- and Salinity-Tolerant Cereal SynCom

A practical DBTL workflow can be illustrated with a hypothetical cereal crop grown in semi-arid soil affected by both drought and salinity. In the Design stage, the target phenotype is defined as improved seedling establishment, root growth, water use efficiency, nutrient acquisition, and yield stability under limited irrigation and moderate salinity. Candidate functional modules may include ACC deaminase activity to reduce stress ethylene, exopolysaccharide production to enhance rhizosphere water retention and soil aggregation, phosphate solubilization to support nutrient acquisition, siderophore production and biocontrol activity to suppress opportunistic pathogens, and osmoprotectant- or antioxidant-associated traits to reduce stress injury [44,45,69].

In the Build stage, candidate strains are selected from stress-adapted rhizosphere or endophytic communities and assembled into a minimal consortium. Compatibility screening is conducted using co-culture assays, antagonism tests, growth kinetics, metabolite profiling, and functional assays. The goal is not to maximize species number but to construct a functionally complementary consortium with limited negative interactions. Strains should also be screened for formulation compatibility, pesticide tolerance, and survival under desiccation, salinity, and temperature fluctuation because field performance depends not only on beneficial traits but also on persistence and ecological compatibility [12,69].

In the Test stage, the consortium is evaluated first under controlled growth-chamber conditions, then in greenhouse soil microcosms, and finally in replicated field plots. Input variables include strain identity, inoculum ratio, carrier type, seed-coating dose, irrigation regime, salinity level, soil type, and crop genotype. Output variables include colonization efficiency, persistence, microbial community shifts, root architecture, photosynthetic performance, stress biomarkers, nutrient uptake, yield components, and water use efficiency. Amplicon sequencing, shotgun metagenomics, targeted qPCR, metabolomics, and root phenotyping can be integrated to link microbial persistence with plant performance [69,79].

In the Learn stage, data from omics, phenotyping, and agronomic measurements are used to refine the consortium. Machine learning models can identify which strain combinations, functional traits, or environmental variables best predict performance. However, scalability remains a major constraint, and laboratory performance may not translate directly to field reliability in native soils. Therefore, DBTL should be implemented as an adaptive framework that tests performance across increasing ecological complexity [10,40].

## 4. Light Synthetic Biology

**Figure 4:**
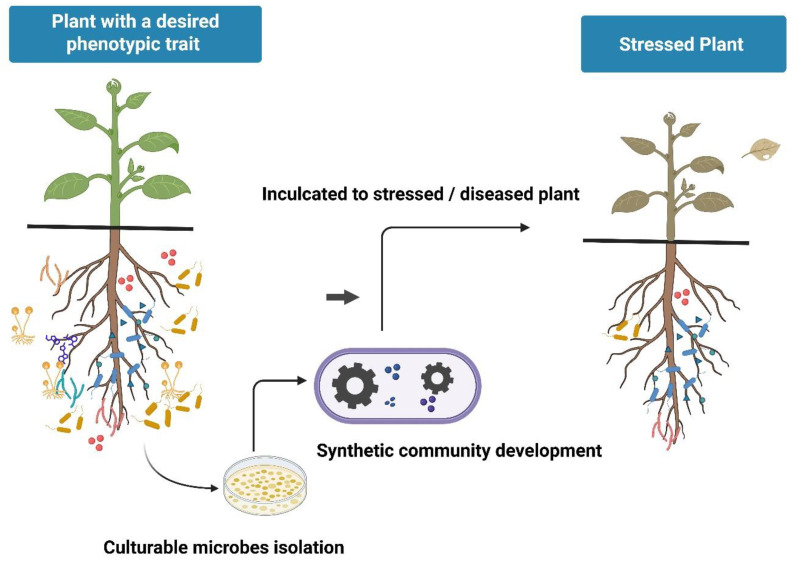
Light synthetic biology toolkit for rhizosphere applications: chassis selection, circuit concepts (quorum sensing, stress toggles, and nutrient valves), and safeguards (biocontainment and orthogonality).

Interest in contemporary biosystems engineering has catalyzed new strategies for designing and developing microbiome structures with specified functionality for targeted applications, such as engineering the plant rhizosphere microbiome to enhance agricultural productivity. In this review, we discuss criteria for choosing microbial chassis and functional circuits, as well as design principles such as biocontainment and orthogonality in microbial systems. Figure 4 summarizes representative chassis options, circuit concepts, and safety safeguards.

### 4.1. Choosing Microbial Chassis

Selection of a microbial chassis is a fundamental consideration in synthetic biology, particularly for agricultural crops. Genetically tractable chassis organisms that are easy to cultivate and beneficial in the rhizosphere, such as *Bacillus*, *Pseudomonas*, *Trichoderma*, and *Azospirillum*, are preferred [12,69]. *Bacillus* species, well known as ideal biocontrol agents, have also been reported to produce antifungal compounds that suppress pathogens [69,80]. Although *Pseudomonas* has a reputation as a dormant saprophytic bacterium, some strains, such as *Pseudomonas syringae*, are also known to promote plant growth [81]. *Trichoderma* fungi enhance plant nutrient uptake through mycorrhizas and provide protection against nematodes and pathogens [69,82]. Ecological compatibility is also important, and selected consortia should remain stable, functional, and beneficially interactive under the intended application conditions.

In addition to *Trichoderma*, *Bacillus*, and *Pseudomonas*, other functional biocontrol organisms may be relevant depending on the target pathosystem. These include entomopathogenic fungi such as *Beauveria* and *Metarhizium*, which are widely recognized as soil-associated insect biocontrol fungi, as well as *nematophagous* fungi such as *Purpureocillium lilacinum* and *Pochonia chlamydosporia*, which have been used against plant-parasitic nematodes and can be relevant in crop protection-oriented microbiome designs [83,84,85]. Although these fungi are not universal components of rhizosphere consortia, they may be appropriate where insect or nematode pressure is a major agronomic constraint.

### 4.2. Circuit Ideas

Synthetic circuits are designed in microbes to couple biological pathways that respond to fluctuating environmental inputs. Quorum sensing (QS), a type of cell–cell communication, coordinates bacterial behavior based on population density [86,87]. Bacteria can also modulate metabolic functions such as biofilm formation, virulence factor expression, or intracellular nutrient uptake dynamics according to the community state in a QS-dependent manner [88,89].

In addition, stress-response toggles can be implemented to activate targeted responses during abiotic stress, thereby enhancing stress resistance in plants. For example, synthetic biology approaches can be used to design gene circuits that regulate stress-responsive phytohormones, enabling microbes to actively contribute to plant health during water stress or nutrient-deficient conditions [89,90]. This can be achieved through so-called nutrient valves, which govern whether important nutrients, especially nitrogen and phosphorus, are absorbed or released. These valves may be engineered to maximize resource use and promote plant development and productivity under nutrient-limited conditions [91].

### 4.3. Biocontainment and Orthogonality

Successfully biocontainment of engineered microbes is important to avoid negative consequences for the natural environment during the use of these microbes. This necessitates orthogonal systems that prevent engineered pathways from interfering with native microbial functions [92,93]. Synthetic biology principles have been used, for example, to design circuits that respond exclusively to synthetic signal molecules not found in natural environments, thereby reducing the risk of crosstalk between engineered and wild-type microbes [94].

These orthogonal circuits may be necessary for programming engineered strains to perform important functions in an ecological niche without altering the cost–benefit ratios that the existing microbiome has evolved to maintain. Additionally, incorporating “kill switches” or dependence on artificial or synthetic nutrients for survival in nature allows for the creation of engineered communities that are safe and controllable [95].

Ultimately, the use of suitable microbial chassis, functional circuits capable of tunable responsiveness to physiological triggers, and biocontainment via orthogonally constructed systems would enable the application of synthetic biology to enhance the functions of microbial consortia that support agricultural processes. Such an integrated strategy would sustainably reinforce agricultural technologies and enhance the resilience of crop production systems.

## 5. Delivery and Formulation for Field Reality

Achieving field-scale outcomes in microbiome engineering requires effective delivery and formulation strategies, including seed coatings, encapsulation, co-formulation with biostimulants, and attention to shelf life and on-farm handling.

Seed coatings and granules are innovative methods for applying microbial inoculants in the field. Coating seeds with beneficial microorganisms is a direct and effective delivery method that enhances germination, growth, and establishment by providing immediate access to the microbial community at sowing. Encapsulation strategies that enclose microbial inoculants in coating materials can protect microbes from harsh environmental conditions and improve their viability [96].

Biochar, due to its porous structure and large surface area, which favor microbial attachment and activity [97], has strong potential as a microbial inoculant carrier. In addition to enhancing the survival of microorganisms within biochar, it helps retain nutrients in the soil, indirectly supporting plant growth [98]. Biochar carriers can also regulate the dynamics of the microbial community, positively influencing soil health and plant growth [99,100].

The effectiveness of microbial products can be significantly increased by combining microbial formulations with biostimulants such as humic substances and micronutrients. Improved oxygen accessibility promotes soil structure development, increases the availability of elemental nutrients, and stimulates beneficial microbial activity [101,102]. In co-formulated microbial inoculants, these substances act synergistically, as demonstrated by increased microbial viability and enhanced plant responses to microbial interactions facilitated by humic substances [103].

Polymers enhance the stability and shelf life of gases in microbial product formulations. They can also be used for microencapsulation, protecting microbes from environmental factors such as temperature and desiccation, thereby improving survival during transport and storage [104]. This multifaceted approach, including co-formulation, is essential for maximizing the potential of microbial inoculants as key components of agriculture.

To ensure microbes remain effective in the field, their shelf life and viability must be maintained during transport and storage. Shelf life is influenced by temperature, humidity, and packaging methods. For formulations sensitive to ambient conditions, cold-chain logistics can help preserve microbial viability [99].

Just as it is important to keep all microbes alive until delivery, on-farm handling practices are also crucial. Comprehensive guidance documents, including recommended storage conditions and handling practices that minimize microbial loss [97], would be valuable for producers. Approaches such as moisture-proof packaging or temperature-controlled transportation can extend shelf life and ensure that active microorganisms are delivered to the field in quantities above a threshold level and in an optimal environment [96,105].

### 5.1. Strain Tracking and Field Persistence

Tracking the survival and persistence of introduced microbial components is essential for distinguishing true inoculant establishment from transient application effects or indirect stimulation of native taxa. Several approaches are available depending on regulatory acceptability and experimental resolution. Culture-dependent re-isolation on selective media is simple but lacks strain-level specificity. Strain-specific qPCR or digital PCR can quantify persistence when unique genomic regions are identified. Amplicon sequencing can monitor broad community shifts but often cannot distinguish closely related inoculant strains. Shotgun metagenomics, genome-resolved metagenomics, whole-genome resequencing, SNP-based markers, and metagenomic read recruitment offer higher resolution for tracking strain persistence and functional potential in complex soil communities [79,106].

In experimental systems, tagged strains, reporter genes, or genetic barcodes can be informative, but their use may be limited by biosafety and regulatory constraints. Therefore, field studies should combine at least one strain-specific detection method with community-level profiling and plant performance measurements to determine whether the inoculant survived, colonized the rhizosphere, altered the resident microbiome, and contributed to the observed phenotype [10,69].

### 5.2. Comparative Assessment of Formulation Technologies for Microbiome-Enabled Field Deployment

Formulation remains a major translational bottleneck in rhizosphere microbiome engineering because microbial viability, shelf life, release dynamics, and compatibility with field practices vary substantially among delivery platforms. Seed coatings, polymer encapsulation, biochar-based carriers, liquid inoculants, and granules each offer distinct operational advantages but also impose specific biological and logistical constraints. To highlight these trade-offs, Table 3 compares the principal formulation technologies currently considered for microbiome-enabled field deployment.

### 5.3. Comparing Intervention Strategies: Single Strains, Synthetic Consortia, and Native Microbiome Steering

Microbiome-based interventions can be broadly divided into three strategies: single-strain inoculation, synthetic consortia, and indirect steering of native microbiomes. Single strains offer simplicity, a lower characterization burden, and easier quality control but often lack robustness under field conditions. Synthetic consortia may provide complementary functions, redundancy, and broader stress buffering, but they are more difficult to optimize, formulate, track, and regulate.

Native microbiome steering, such as crop rotation, host genotype selection, exudate modulation, or soil amendments, avoids the need to establish introduced strains but provides less precise control over microbial composition. No strategy is universally superior: single strains may suit narrowly targeted functions, synthetic consortia may suit multi-trait challenges, and native microbiome steering may suit low-input ecological intensification. A key challenge is determining which strategy offers the best balance of controllability, cost, and field reliability under specific agronomic conditions.

## 6. Multi-Omics and Phenotyping Pipelines

Combining multi-omics strategies with high-throughput, high-resolution imaging provides insight into complex plant–microbe interactions and plant health while enabling a phenomic perspective on sustainable agriculture. This section discusses measurement methods, from metagenomics to metatranscriptomics, together with phenotyping systems that link laboratory and field observations.

Approaches such as 16S rRNA gene sequencing, Internal Transcribed Spacer (ITS) sequencing, and shotgun metagenomics are the main methods for characterizing the rhizospheric modular structure of microbial consortia. These techniques provide improved insights into community dynamics and functions by enabling accurate identification and quantification of taxa, as well as strain tracking [107]. Shotgun metagenomics, in particular, reveals the functional potential of the microbial community and allows high-resolution mapping of gene clusters associated with beneficial traits, such as nutrient uptake or pathogen inhibition [108]. Using these methods, microbial community compositions can be linked to plant phenotypes, implicating specific microbial strains in strategies that benefit plant health and yield.

Adding metatranscriptomics and metabolomics to current phenotyping pipelines should be implemented to provide a more complete picture of microbial activity and interactions with plants. Metatranscriptomics, the study of mRNA within microbial communities, reveals functional gene expression and offers valuable information about metabolic potential and symbiotic interactions with the host plant [109]. Similarly, metabolomics provides access to the small-molecule profiles of plant and microbial metabolites, including exometabolites. These exometabolites can indicate their roles in plant–microbe interactions, especially root exudates, and may, in some cases, reflect plant stress or health [110].

Collectively, these multi-omics approaches provide a systems biology perspective for revealing the complex and interdependent biochemical networks operating in the rhizosphere. These include gnotobiotic, dietary-controlled, and agnosome approaches that couple metabolomic and transcriptomic data to resolve pathways involved in nutrient acquisition, stress response, and plant immune signaling. This knowledge enables the targeted use of biostimulants to enhance crop stress tolerance [111].

Rhizoboxes, rhizotrons, and, more recently, microfluidic devices have introduced new strategies for measuring and observing the root systems of various species and their interactions with soil microbes in innovative experimental designs [112]. These systems are designed for researchers to study how defined microbial consortia influence root morphology, nutrient assimilation, and whole-plant physiology in controlled environments [113].

Furthermore, establishing connectivity between greenhouse and field applications is a critical step in translating discoveries to real-world field conditions. These high-throughput phenotyping systems enable testing of microbial inoculants under various environmental settings, supporting the improvement of product formulation and application strategies in agriculture [111].

This approach enables the linking of plant phenotypes to interdependence within microbial communities in the same ecological niche through plant–microbe interaction network analysis. Such multi-omics data can establish relationships between plant health and key microbes [114]. Causality studies distinguish correlation from direct effects by exchanging samples between plants grown with specific microbes and corresponding controls [115], allowing scientists to identify the functional importance of certain microbes or metabolites within the broader network of plant–microbe interactions.

Shifts in the relative abundance of microbes within assemblages or changes in their functional capabilities may impact plant growth, but the extent is often unknown [116]. A model-driven approach will help develop improved management practices and support the rational design of microbial consortia to address specific agronomic challenges.

Integrating multi-omics with advanced phenotyping technologies will help map links between microbial populations and plant phenotypes and support the development of targeted interventions for sustainable agriculture.

### Quantitative Indicators for Microbiome Engineering Success

Predictive microbiome engineering requires quantitative indicators that link community structure to function. Useful metrics include colonization frequency, persistence half-life, relative abundance of introduced strains, yield response ratio, water use efficiency, nutrient use efficiency, disease suppression index, and stability across sites or seasons. Ecological metrics such as alpha and beta diversity, functional redundancy, network centrality, modularity, keystone score, interaction strength, and resilience after disturbance can also be used. Co-occurrence network analysis has been widely used to infer potential microbial interconnections and assess community stability through topological properties, although such networks should be interpreted cautiously because correlation does not prove direct interaction [39,69].

A practical success index may combine three components: establishment, function, and robustness. Establishment indicates whether introduced strains persist in the rhizosphere; function indicates whether target traits such as nutrient acquisition, ACC deaminase activity, EPS production, or pathogen suppression are expressed; and robustness indicates whether the benefit is maintained across soil types, crop genotypes, stress levels, and seasons. Such semi-quantitative frameworks can help distinguish deterministic design from statistical steering and guide decision-making before field-scale deployment [10,12].

## 7. Precision Agriculture Interfaces

With the demand for technology that can collect and analyze information about soil and crop health, the development of precision agriculture is advancing. We highlight major players in this field, including field sensors, proximal sensing via UAVs, variable-rate applications of microbial inputs, and crop–microbiome interaction digital twins.

Field sensors are a crucial component of precision agriculture, providing real-time feedback on ambient conditions. Key variables to measure include soil moisture, electrical conductivity (EC), nitrate, and canopy temperature, all of which are critical for irrigation and fertilization [117,118]. For example, soil moisture biosensors can improve irrigation scheduling by optimizing water use while maintaining soil health [119]. Similarly, electrical conductivity sensors provide information about nutrient availability in the soil, while nitrate sensors are essential for managing nitrogen and preventing environmental pollution [118]. Recent advances in wireless sensor network (WSN) technology have further enabled long-distance monitoring of these parameters, allowing farmers to make informed adjustments to their cropping systems [120].

The use of drones equipped with multispectral and thermal sensors has transformed agricultural data collection. Large-scale aerial monitoring with drones provides essential information, with flying height, drone orientation, and flight speed having the greatest influence on crop growth and thermal attributes [121,122]. Multispectral imaging offers valuable data on environmental parameters by measuring spectral reflectance, thereby revealing plant health and nutrient deficiencies [123]. Thermal sensors can detect plant water stress, which can then inform irrigation schedules and improve crop water use efficiency [124]. Combining terrestrial data with drone data creates an optimal foundation for agricultural decision-making.

Variable-rate technology (VRT) enables site-specific application of inputs such as fertilizers, pesticides, and microbial inoculants based on field variability. With sensor data and prescription maps, beneficial inputs can be precisely applied to areas of the field that are biochemically or biophysically responsive, thereby increasing efficiency and minimizing waste [125]. This approach enhances overall agricultural productivity and benefits the environment by reducing agrochemical overuse [122]. Studies have shown that the advantages of improved plant resistance and metabolic pathways achieved through VRT-applied microbial inoculants outweigh the risks of adverse environmental impacts [126].

Digital twin technology in agriculture is an innovative tool that allows farmers and researchers to create a virtual replica of a real-world agricultural system. Crop digital twins can integrate real-time sensor data with predictive modeling to simulate crop–microbiome interactions across various environments [127]. This technology enables users to understand the effects of different parameters on plant and microbial health and to test scenarios for decision-making [128]. In precision agriculture, digital twins facilitate simulations of multiple “what-if” scenarios, allowing farmers to manage and allocate resources and interventions more effectively in response to predicted conditions [129].

In summary, the integration of advanced sensors, drone technology, variable-rate applications, and digital twins makes farms more productive, environmentally friendly, and resource-efficient. Together, these tools enable farmers to make decisions that address the complexity of relationships within their agricultural systems.

## 8. AI/ML for Microbiome Engineering

The synergy between artificial intelligence (AI) and microbiome engineering offers new opportunities to enhance food production, efficiency, and sustainability through microbiome-mediated improvements in agriculture. The promise of this approach lies in the ability to design plant growth- and health-associated microbial consortia in real time, based on a comprehensive understanding of plant–microbe interactions using machine learning and active learning methods. This integrated knowledge of plant-beneficial microbiomes is highly relevant, as it may eventually enable the design of plant-associated microbial consortia with engineered functions that promote growth and health. However, interactions among species at various levels of biological organization complicate collective learning.

Feature engineering is a crucial step in machine learning applications involving multi-omics data. Combining omics data (genomics, transcriptomics, metabolomics, proteomics, and phenomics) with agronomic data provides a holistic view of plant–microbe relationships [130,131]. Microbial diversity indices, functional gene profiles, and plant stress markers are particularly useful features that can enhance the predictive power of microbiome engineering models. Integrating sequencing technologies with less explored experimental aspects, such as plant characteristics and microbial functions, holds great potential for selecting effective strains and optimizing consortium structure [132] and depends on the development of computational frameworks that support such integration.

In recent years, several machine learning models have been applied to select microbial strains and optimize their proportions in consortia. Machine learning algorithms analyze omics data (gene expression, metabolomics, etc.) and agronomic datasets to identify patterns, hidden relationships, and associations between specific strains and beneficial traits (e.g., stress tolerance or nutrient availability) [133]. Some models can even optimize microbial mixtures based on functional roles, ensuring that functional representation is sufficient to meet specific agronomic targets [134]. These computational approaches accelerate the discovery of functional microbial consortia, reducing reliance on trial-and-error laboratory methods.

The use of active learning and Bayesian optimization methods is essential for accelerating the (DBTL) cycles in microbiome engineering. With active learning, researchers can iteratively and intelligently query the most informative data points, leading to improved model performance with fewer experimental rounds [131]. Pairing these methods with Bayesian optimization enables iterative exploration of the parameter space to incrementally refine the microbial community in the most information-theoretically optimal way [134]. This cycle allows for rapid design and redesign of synthetic microbial consortia tailored to specific environments or stressors and enables continuous improvement of these consortia.

AI analytics help agronomists make real-time decisions using decision dashboards. Dashboard visualizations of processed and integrated data sources, including measured signals from sensors and multi-omics analyses, provide field-level insights for agronomists to assess microbial performance in the field [133]. Given the demand for rapid, actionable insights into the efficacy of microbes and their effects on soil health and plant responses, decision dashboards are being developed to guide farmers’ management decisions for optimal agricultural outcomes with minimal effort [10]. Predictive analytics can also simulate potential scenarios based on alternative methodologies, helping to reduce the gap between planning and implementation.

Thus, the AI framework serves as a tool for microbiome engineering that could expand the role of microbiome-based climate-smart agriculture. Through feature engineering, machine learning models, active learning strategies, and decision-support tools, we can enhance the efficiency of microbial consortia to be more crop-sustainable and climate-resilient in response to soil and environmental changes.

## 9. Stress Targets and Use Cases

When rhizosphere microbiome engineering is explicitly linked to clear performance targets, such as stress alleviation under drought, salinity, temperature extremes, and biotic threats, as well as measurable agronomic endpoints like stand establishment, water use efficiency, yield stability, and disease suppression, it becomes “use-case driven.” Under these stresses, beneficial microbes often act through overlapping mechanisms rather than single pathways: (i) enhanced root system architecture and resource acquisition, (ii) amelioration of oxidative and osmotic damage, (iii) hormonal rebalancing (ABA/IAA/ethylene), and (iv) priming of immune responses [135,136,137]. As a result, stringent approaches often use multi-trait strains or small consortia that provide complementary functions, thereby mimicking field heterogeneity [137,138].

### 9.1. Drought Resilience

Water deficit causes low relative water content, inhibits photosynthesis, and increases reactive oxygen species (ROS) in plants, while soil drying affects diffusion processes, nutrient availability, and the physical structure of the rhizosphere. Drought resilience-linked microbiome interventions that have been repeatedly associated with improvements at the root–soil interface (aggregation, biofilm formation, rhizosphere hydration) combined with physiological reprogramming of the plant (osmolyte production, antioxidant capacity, and stomatal regulation) [135,137].

However, drought-tolerant plant growth-promoting rhizobacteria (PGPR) and endophytes offer unique benefits; through phytohormone production and signaling cross-talk, these beneficial microbes influence root morphology and architecture (such as root length, branching, and functional surface area) to utilize spatially heterogeneous soil water [135,136]. Similarly, drought-sensitive microbes can enhance osmotic adjustment and stimulate antioxidant enzyme activities, leading to reduced membrane damage and greater genetic and metabolic stability in plants under water deficiency [136,137,138].

The roles of exopolysaccharides (EPS) and biofilms—traits relevant to drought due to their stabilization of soil aggregates, enhancement of microscale water retention, and buffering against rapid drying–rewetting cycles [137,139]—are frequently highlighted at the rhizosphere scale. Ethylene is another drought-related factor: stress increases ethylene, which inhibits root growth. For example, microbes with ACC deaminase activity reduce the pool of ethylene precursors, providing a means to maintain developmental and stress tolerance pathways associated with root growth during periods of water deficit [135,140]. In line with these mechanisms, recent syntheses highlight that the performance of drought inoculants is more consistent when inoculants provide different, complementary traits (such as EPS production, hormone modulation, and nutrient acquisition) and when evaluation is conducted under compound stresses typical of those expected in production systems [137,138].

### 9.2. Salinity

Combined osmotic stress (reduced water supply) and ionic stress (Na^+^ toxicity and disruption of K^+^/Na^+^ homeostasis) from salinity and sodicity lead to downstream effects on photosynthesis, enzyme activity, and ultimately reproduction. Importantly, established proxies such as “Na^+^ exclusion” do not reliably predict salinity tolerance, as tolerance can be tissue-specific, developmentally conditional, and dependent on traits like biomass reallocation that are not captured by single-trait measures [141]. This complexity aligns with microbiome engineering strategies that address multiple bottlenecks simultaneously.

Soil salinization and the salinization of agricultural soils, particularly under climate change and anthropogenic influences, are likely to become globally widespread and persistent. Consequently, salt-adapted microbes and PGPR may improve plant performance by balancing osmotic regulation, facilitating nutrient acquisition (especially K^+^ and P), modulating stress-responsive gene expression, and enhancing antioxidant systems [142,143]. Among EPS-producing rhizobacteria, EPS remains an important trait for saline soils, as the polymer is known to improve soil structure and rhizosphere water availability and has also been associated with ionic balance under stress conditions [139]. ACC deaminase-positive PGPR are similarly often implicated in salinity mitigation by reducing stress ethylene and enhancing physiological stability [140,143]. Because many arid and coastal systems experience combined drought–salinity events, use-case evaluation should explicitly test inoculants under compounded stress when possible [136,143].

### 9.3. Heat and Cold

Temperature extremes, through ROS bursts and impaired enzymatic kinetics, disrupt membranes, photosystems, protein folding, and metabolic homeostasis. Microbial strategies that promote thermotolerance often correlate with enhanced antioxidant capacity and ROS detoxification, modulation of phytohormone signaling, sustained photosynthetic performance, and priming of stress-responsive gene networks [144]. Reviews on microbe-mediated mitigation of heat stress highlight that endophytes and PGPR confer heat tolerance through cumulative physiological and molecular mechanisms, reinforcing the view that thermotolerance should be studied and addressed as a systems phenotype rather than a linear pathway [144]. Experimental work with endophytic bacteria associated with rice further supports this functionality, showing that these bacteria can be introduced into relevant strains to enhance performance under heat stress [145].

Initially, cold stress induces oxidative stress, metabolic reduction, and transcriptional reprogramming in a manner similar to heat stress. For example, recent studies in tomato seedlings demonstrate that cold-tolerant bacteria alleviate cold stress by fully activating transcriptional networks involved in ROS homeostasis, cold-stress signaling, and hormone pathways, consistent with a “priming plus metabolic rescue” model of cold stress resilience [146]. These findings support the idea of tailored screening, where candidate lines are evaluated not only for low-temperature growth promotion but also for their effects on oxidative stress markers and cold-responsive expression signatures [139,146].

### 9.4. Biotic Stress

Rhizosphere engineering for biotic threats seeks to decrease the incidence and severity of disease by a combination of direct antagonism (antibiotics, lytic enzymes, niche competition) and host immune priming, often presented as induced systemic resistance (ISR) [147]. Recent syntheses endorse ISR, as a multi-partner event molded by plant–microbe–pathogen interactions, microbial signal timing, and community composition, and emphasize that biotic “use cases” should be defined by pathosystem and growth stage, not generic pathogen categories [147].

While direct suppression of microbial growth and immune activation represent potentially different organismal outcomes, bacterial cyclic lipopeptides and related secondary metabolites provide a mechanistic bridge between these fates by functioning as both antimicrobials and elicitors of plant immunity and systemic resistance [148]. *Bacillus* spp. are commonly emphasized for their capabilities in lipopeptide production (e.g., iturin, fengycin, and surfactin families) with known connection to rhizosphere competence and pathogen suppression [149].

The plant also plays an active role: modification of root exudation under pathogen attack can reshape rhizosphere communities into suppressive states by either attracting or repelling soil microbes, a pattern increasingly supported across plant systems (“cry for help”). The more interesting claims from a use-case perspective are those that (i) connect a defined microbial trait or metabolite class (e.g., lipopeptides, ISR triggers, EPS/biofilms) with (ii) a measurable host or disease endpoint (immune markers, disease index reduction, yield protection), and (iii) include translation bottlenecks (colonization stability, context dependence, and formulation variability) [136,147].

Climate-smart microbiome engineering should explicitly address multi-stressor environments rather than single-stress models. In field systems, drought, salinity, heat, nutrient limitation, and pathogen pressure often occur simultaneously or sequentially, and climate volatility can alter soil moisture, aeration, nutrient diffusion, microbial metabolism, and root exudation patterns. During drought, plant microbiomes may shift toward taxa adapted to water limitation, while genome-resolved studies have linked drought-induced rhizosphere shifts to microbial traits such as iron metabolism and stress-associated functional potential [79]. Under disease pressure, plants can alter microbiome assembly and recruit protective microbial functions, but these effects are influenced by soil type, plant genotype, and resident community structure [39]. Therefore, engineered microbiomes should be considered robust only when they maintain establishment and function across realistic stress combinations, whereas narrowly optimized consortia may remain fragile under climate volatility.

## 10. Field Trials, KPIs, and Economics

The term “Adaptive Field Trials,” as used in this document, applies to the following stage of adaptive learning: field experiments. After new agricultural practices or technologies have been evaluated in controlled experiments or other set circumstances, they must be tested under real-life conditions through adaptive field trials, where their effectiveness and economic viability can be assessed. This section focuses on trial design, agronomic KPIs, cost–benefit analyses, and agricultural environmental impacts.

Field trials can generate the necessary data on the expected benefits of emerging agricultural interventions; therefore, proper design is essential to obtain robust results. Split-plot design is a factorial test design that enables simultaneous testing of one or more treatments while conserving resources and effectively comparing two factors, such as treatment type and application method [150]. To evaluate the effectiveness of agricultural innovations across various environmental conditions and soil types, multi-site trials are required to yield results that are more relevant to different regions [151]. Additionally, long-term data collection provides a comprehensive view of the interactions between climate and agriculture, which determine the extent to which sustainability can be addressed [152]. Impacts on yield and soil health are revealed only through year-to-year interactions.

In agriculture, KPIs associated with appropriate interventions are used to determine whether effective interventions have been implemented. Yield, a basic and fundamental KPI, reflects the number of product items produced per unit area [153]. Water use efficiency (WUE) is commonly defined as grain yield (or biomass) produced per unit of water used by the crop and is a key KPI in water-limited environments [154]. Nutrient use efficiency (NUE), the ability of crops to efficiently utilize supplied nutrients, is a key component of sustainable and economically viable agriculture [155]. Therefore, evaluating yield stability across seasons to ensure consistency from one year to another is essential for practitioners. Understanding climatic limits is important for planning and risk management in cropping systems [156].

A detailed Cost Benefit Analysis (CBA) is needed to assess the economic viability of agricultural technologies or practices from the perspectives of farmers and other stakeholders [157]. CBA weighs both direct and indirect costs against benefits to support better policy decisions. Furthermore, risk assessment helps farmland end users identify uncertainties and plan for adverse conditions, enhancing resilience to climate change and business fluctuations [158].

As the global market advances toward greater environmental friendliness, carbon and water footprints in agriculture are becoming increasingly important [99]. Consumer trends may favor more sustainable practices that reduce carbon and water footprints [159]. Quantifying these impacts can help establish a strong business case for adopting sustainable practices while contributing to global sustainability goals.

**Figure 5:**
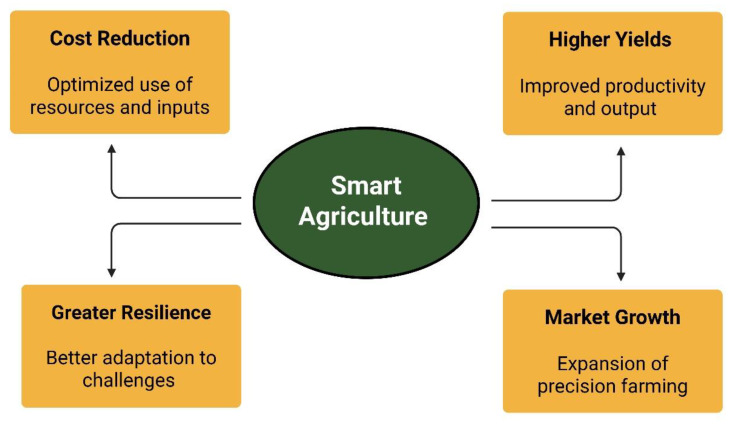
Field validation pipeline for microbiome-enabled inputs: trial design (multi-site/multi-year), agronomic KPIs, cost–benefit and risk analysis, and environmental footprint assessment.

Trial design, KPI selection and monitoring, cost–benefit and risk analyses, and ecological footprint assessment are critical tools for achieving success in agricultural innovation. These principles help researchers and practitioners maximize agricultural production in an environmentally sustainable and cost-effective manner. They are informed by experiences from both smallholder and large-scale farming systems. Figure 5 summarizes this process, providing a practical pathway from field trial design through KPIs to economic evaluation. Currently, the field performance of microbiome interventions should generally be considered variable and site-dependent rather than reliably transferable across soils, climates, and host genotypes.

### 10.1. Field Inconsistency, Regulation, and Commercialization

Despite the promise of SynComs and microbiome-enabled inputs, field performance remains inconsistent. Common reasons include poor survival after formulation, inadequate rhizosphere colonization, displacement by native taxa, mismatch between laboratory screening conditions and field stress combinations, and temporal variability in soil moisture and temperature. Ecological drift, horizontal gene transfer, and invasion by resident microbial populations may further reduce predictability, especially when introduced strains are applied to highly diverse native soils. Therefore, field validation should report not only crop responses but also inoculant persistence, community shifts, and site-specific environmental variables [40,69].

Regulatory and commercialization barriers must also be considered. Microbial products must meet safety, identity, quality control, and efficacy requirements. For genetically engineered microbes, additional biosafety concerns include unintended persistence, gene flow, non-target effects, and containment. Even for non-engineered strains, commercialization requires stable formulation, acceptable shelf life, compatibility with seed treatments and pesticides, scalable production, farmer usability, and economic advantages over conventional agronomic practices. Cost–benefit evaluation should therefore compare microbiome-based products with standard fertilization, irrigation, and crop protection programs rather than evaluating them in isolation [10,40,160].

### 10.2. Constraints, Risks, and Failure Modes in Rhizosphere Microbiome Engineering

Despite rapid progress, rhizosphere microbiome engineering is still constrained by major biological, technical, and translational risks. Engineered consortia may fail because introduced strains do not persist, are outcompeted by native taxa, or express target traits only under limited environmental conditions. Functional performance may decline when inoculants are transferred from controlled systems to heterogeneous field soils. Additional risks include formulation instability, short shelf life, compatibility issues with seed treatments or agrochemicals, uncertain dose–response relationships, and variable responses across cultivars, soil types, and seasons. For engineered strains, regulatory scrutiny, biosafety assessment, and non-target ecological effects impose further constraints. These limitations indicate that current microbiome engineering should be considered a context-dependent, probabilistic intervention rather than a universally reliable technology.

### 10.3. Rhizosphere Microbiome Engineering Within Climate-Smart Agriculture

Rhizosphere microbiome engineering is especially relevant to climate-smart agriculture because it supports the three core CSA pillars: sustaining productivity, strengthening resilience and adaptation, and reducing environmental impacts where possible. For productivity, engineered rhizosphere functions may improve nutrient acquisition, plant establishment, and yield stability. For adaptation, microbiome-based strategies can enhance tolerance to drought, salinity, temperature extremes, and biotic stress. For mitigation, microbiome-informed interventions may improve fertilizer-use efficiency, reduce nutrient losses, influence nitrous oxide-related nitrogen cycling, and contribute indirectly to soil carbon stabilization through improved root growth, aggregate formation, and rhizosphere carbon turnover [69,79,160].

**Figure 6:**
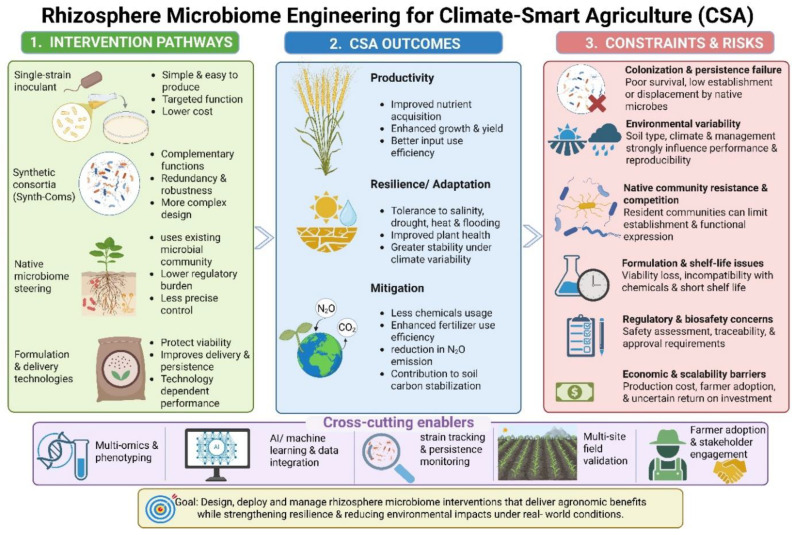
Rhizosphere microbiome engineering for climate-smart agriculture.

However, mitigation-related outcomes should be interpreted cautiously, as direct long-term field evidence linking engineered rhizosphere consortia to lower N_2_O emissions or durable carbon sequestration remains limited [10,40]. Therefore, rhizosphere microbiome engineering should not be viewed solely as a plant growth promotion strategy, but as a potential component of broader climate-smart crop management systems (Figure 6).

## 11. Conclusions and Outlook

Rhizosphere microbiome engineering is evolving from descriptive microbiome science to a more intervention-oriented discipline, but its practical value will depend on its ability to deliver reliable functions under field heterogeneity. The central message of this review is that progress in this field should be viewed not as the accumulation of isolated technologies, but as the integration of linked stages in a translational pipeline: ecological understanding of rhizosphere assembly, rational consortium design, lightweight synthetic biology, formulation and delivery, multi-omics and phenotyping, AI-enabled optimization, and field validation against agronomic and climate-smart endpoints. Throughout this pipeline, the strongest conclusion is not that microbiome engineering is already predictable, but that it is becoming increasingly designable under constrained, testable, and context-dependent conditions [10,12,40].

For climate-smart agriculture, the most credible near-term value of rhizosphere microbiome engineering lies in improving stress resilience, nutrient use efficiency, and management precision rather than assuming universal mitigation benefits. Microbiome-based interventions may enhance fertilizer-use efficiency, stabilize crop performance under drought or salinity, and, in some contexts, alter nitrogen and carbon cycling. However, direct evidence linking engineered rhizosphere systems to durable reductions in N_2_O emissions or long-term soil carbon sequestration remains limited and should be interpreted cautiously. Thus, the climate-smart potential of microbiome engineering is strongest in adaptation and input-efficiency pathways, while mitigation-oriented claims are still emerging [79,160].

This review also argues that single strains, synthetic consortia, and native microbiome steering should be viewed as complementary tools rather than competing approaches. Single strains offer simplicity and easier quality control but often lack robustness in the field. Synthetic consortia may provide functional complementarity and redundancy but are harder to optimize, formulate, track, and regulate. Native microbiome steering through crop rotation, host genotype, soil amendments, or exudate-mediated recruitment may be less precise, yet in some agroecosystems it may offer greater feasibility and persistence. Therefore, the key translational challenge is not to identify one universally superior strategy, but to match the intervention type to the crop–soil–climate context and to the level of controllability that is realistically achievable [37,38,69].

Several priorities emerge for the next phase of the field. First, microbiome-based products must be supported by formulation technologies that preserve viability, compatibility, and on-farm usability. Second, strain tracking and persistence assessment should become standard so that agronomic effects can be linked to actual inoculant establishment rather than assumed presence. Third, quantitative criteria for success should be adopted, including establishment, function, and robustness across environments. Fourth, multi-site and multi-year validation is essential for distinguishing scalable performance from context-specific effects. Finally, claims regarding climate mitigation, resilience, and economic value should be benchmarked against conventional agronomic practices rather than discussed in isolation [10,40,69].

In this sense, the future of rhizosphere microbiome engineering depends not on microbial complexity alone, but on disciplined integration across ecology, agronomy, formulation science, biosafety, and decision support. The field is most likely to advance by shifting from broad claims of controllability to evidence-based, comparative, and field-realistic design. Under this framework, rhizosphere microbiome engineering can become a credible component of climate-smart agriculture, particularly where resilience, nutrient efficiency, and adaptive management are prioritized.

## Figures and Tables

**Table 1:** Representative summary of the technologies and strategies used in rhizosphere microbiome engineering and precision agriculture.

Category	Technology/Strategy	Purpose	Data or Output Generated	Application Stage
**Microbial design**	Synthetic microbial consortia	Combine complementary microbial functions	Community composition, functional profiles	Design/Build
**Microbial optimization**	Adaptive laboratory evolution (ALE)	Enhance stress tolerance and persistence	Growth rates, stability metrics	Build
**Formulation**	Seed coating	Improve early root colonization	Colonization efficiency	Deployment
**Formulation**	Encapsulation (biochar, polymers)	Protect microbes and control release	Survival rate, release kinetics	Deployment
**Production**	On-farm bioreactors	Local large-scale inoculum production	Cell density, cost efficiency	Deployment
**Phenotyping**	Rhizoboxes and microfluidics	Observe root–microbe interactions	Root traits, colonization patterns	Test
**Omics analysis**	16S/shotgun metagenomics	Community structure and functions	Taxa abundance, gene pathways	Test
**Omics analysis**	Metatranscriptomics and metabolomics	Functional activity	Expression levels, metabolites	Test
**Precision agriculture**	Soil and plant sensors	Monitor moisture, nutrients, stress	Real-time field data	Test/Learn
**Precision agriculture**	Drones and proximal sensing	Spatial crop health assessment	NDVI, thermal indices	Test/Learn
**Data integration**	Digital twin models	Simulate crop–microbiome systems	Predictive scenarios	Learn
**AI and ML**	Strain selection and mix optimization	Improve consortium performance	Optimized microbial combinations	Learn

**Table 3:** Comparison of major formulation technologies for microbiome-enabled field deployment.

Technology	Advantages	Limitations	Best-Fit Application	Risks
**Seed coating**	Direct root-zone delivery; simple integration with sowing	Limited shelf life; compatibility issues with seed treatments	early root colonization	viability loss during storage
**Polymer encapsulation**	Protects microbes; controlled release	added cost; scale-up complexity	sensitive strains; harsh environments	release inconsistency
**Biochar carrier**	high surface area; supports adhesion and moisture buffering	variable quality; inconsistent carrier properties	dry soils; stress-prone systems	non-standardized performance
**Liquid inoculant**	easy application; flexible dosing	short shelf life; lower field persistence	nursery or immediate application	rapid decline in viability
**Granules**	easier field handling; localized placement	may need a higher inoculum dose	field-scale deployment	uneven placement

## Data Availability

No new data were created or analyzed in this study. Data sharing is not applicable to this article.
